# Disparities in Diagnostic Timeliness and Outcomes of Pediatric Appendicitis

**DOI:** 10.1001/jamanetworkopen.2023.53667

**Published:** 2024-01-25

**Authors:** Kenneth A. Michelson, Richard G. Bachur, Shawn J. Rangel, Jonathan A. Finkelstein, Michael C. Monuteaux, Monika K. Goyal

**Affiliations:** 1 Division of Emergency Medicine, Ann & Robert H. Lurie Children’s Hospital of Chicago, Chicago, Illinois; 2 Division of Emergency Medicine, Boston Children’s Hospital, Boston, Massachusetts; 3 Department of Surgery, Boston Children’s Hospital, Boston, Massachusetts; 4 Department of Health Systems Science, Kaiser Permanente Bernard J. Tyson School of Medicine, Pasadena, California; 5 Division of Emergency Medicine, Department of Pediatrics, Children’s National Hospital, George Washington University, Washington, DC

## Abstract

This cohort study compares rates of delayed diagnosis and complications of appendicitis by race and ethnicity and Child Opportunity Index among children in 8 states.

## Introduction

Delayed diagnosis of appendicitis is associated with a high risk of complications.^1^ Disparities in diagnostic timeliness may place children in racial and ethnic minority groups at risk of complications. We compared rates of delayed diagnosis and complications of appendicitis by race and ethnicity and Child Opportunity Index (COI) among children in 8 states.

## Methods

This retrospective cohort study included children younger than 18 years diagnosed with appendicitis who visited any emergency department (ED) in Arkansas, Florida, Georgia, Iowa, Maryland, Nebraska, New York, or Wisconsin between January 1, 2014, and December 31, 2019. We used the Healthcare Cost and Utilization Project State Inpatient and ED databases. The Lurie Children’s Hospital institutional review board approved the study, with an informed consent waiver because patients were anonymized. We followed the STROBE guideline.

Children with a first diagnosis of appendicitis were identified using diagnosis codes. The outcome was probable delayed diagnosis using a validated approach that detects delays using administrative data.^2^ Secondary outcomes were appendicitis complications, including intensive care unit hospitalization, perforated appendicitis, abdominal abscess drainage procedure, bowel resection, multiple abdominal surgeries, and sepsis. The coprimary exposures were race and ethnicity (identified in database; Asian or Pacific Islander, Hispanic, non-Hispanic Black [hereafter, Black], non-Hispanic White [hereafter, White], and other [not further specified]) and area-level COI quartile derived from the patient’s zip code.^3^ More details are given in the eMethods in Supplement 1.

To examine the association of race and ethnicity and COI with delayed diagnosis of appendicitis, we used a mixed-effects logistic regression model with the outcome of delay and a random intercept for ED, including race and ethnicity, COI, age, sex, insurance, and complex chronic condition as covariates. A separate logistic regression model was developed with complications as outcome, including the same covariates plus delayed diagnosis. Data were analyzed from May 17, 2022, to December 5, 2023, using R, version 4.3.0.

## Results

We analyzed 93 136 patients in 997 EDs (60.2% boys; 39.8% girls; mean [SD] age, 11.8 [3.6] years). Delayed diagnosis occurred in 3413 patients (3.7%). Asian or Pacific Islander (adjusted odds ratio [AOR], 1.46; 95% CI, 1.08-1.71), Black (AOR, 1.27; 95% CI, 1.11-1.46), and Hispanic (AOR, 1.15; 95% CI, 1.03-1.29) race and ethnicity were independently associated with delay (Table); the COI was not.

**Table.** Rates of Delayed Diagnosis According to Race and Ethnicity and Child Opportunity Index^a^

Covariate	Risk of delayed diagnosis		Risk of complications	
	Patients, No./total No. (%)	AOR (95% CI)	Patients, No./total No. (%)	AOR (95% CI)
Delayed diagnosis				
No	NA	NA	18 184/89 723 (20.3)	1 [Reference]
Yes	NA	NA	1364/3413 (40.0)	3.40 (3.12-3.69)
Race and ethnicity				
Asian or Pacific Islander	107/2436 (4.4)	1.36 (1.08-1.71)	582/2436 (23.9)	1.18 (1.06-1.32)
Hispanic	889/20 312 (4.4)	1.15 (1.03-1.29)	4712/20 312 (23.2)	1.23 (1.17-1.29)
Non-Hispanic Black	394/7902 (5.0)	1.27 (1.11-1.46)	1842/7902 (23.3)	1.26 (1.17-1.35)
Non-Hispanic White	1563/47 442 (3.3)	1 [Reference]	8667/47 442 (18.3)	1 [Reference]
Other^b^	240/7188 (3.3)	0.96 (0.81-1.13)	1728/7188 (24.0)	1.21 (1.13-1.30)
COI^c^				
<25	835/18 024 (4.6)	1.14 (0.97-1.34)	4362/18 024 (24.2)	1.18 (1.10-1.26)
25 to <50	1009/26 663 (3.8)	1.04 (0.90-1.20)	5635/26 663 (21.1)	1.14 (1.07-1.21)
50 to <75	895/25 090 (3.6)	1.07 (0.94-1.23)	4838/25 090 (19.3)	1.06 (1.00-1.12)
75 to 100	501/17 417 (2.9)	1 [Reference]	3348/17 417 (19.2)	1 [Reference]

Abbreviations: AOR, adjusted odds ratio; COI, Child Opportunity Index; NA, not applicable.

^a^ Models were controlled for age, sex, primary payer, and complex chronic condition.

^b^ Not further specified from the data supplier.

^c^ Scores range from 1 to 100, with higher scores indicating greater opportunity.

Race and ethnicity, COI, and delayed diagnosis were independently associated with complications of appendicitis (Table). Asian and Pacific Islander, Black, Hispanic, and other race and ethnicity were associated with higher risk compared with White race and ethnicity; compared with the highest COI quartile, lower quartiles were associated with complications.

## Discussion

There are several possible reasons for disparities in timely diagnosis of appendicitis. Clinicians may treat children differently by race and ethnicity. Wait times have been shown to be greater and use of analgesia lower for Black and Hispanic children, possibly reflecting clinician perception of a lower likelihood of appendicitis.^4^ The likelihood of appendicitis may be overestimated in White children, leading to timely diagnosis. The timing of presentation may vary by race, ethnicity, and COI. Earlier presentation would tend to increase the risk of delayed diagnosis because symptoms are more subtle.^5^ Excessively late presentation may make diagnosis challenging, as symptoms of complicated appendicitis are often vague. White children and those from areas with higher COI may have access to EDs with more imaging and other resources. Language proficiency varies by race and ethnicity, with limited English proficiency being associated with increased likelihood that a clinician will fail to elicit findings compatible with appendicitis.^6^

Study limitations are the lack of clinical information and ecological measure of socioeconomic status. Delayed diagnosis, race, ethnicity, and COI were independently associated with complications, suggesting that diagnostic timeliness does not fully explain outcomes disparities. Improving appendicitis disparities will depend on improving diagnostic approach and advancing health care equity more broadly. Efforts to reduce disparities are warranted, including evaluation and implementation of equity-focused clinical pathways.

## Notes

Supplement 1. **eMethods.** Exposures and Outcomes in Study Population
eReferences.

Supplement 2.
Data Sharing Statement
