# Supplementary material for: Disparities in Diagnostic Timeliness and Outcomes of Pediatric Appendicitis
Source: JAMA Netw Open. 2024 Jan 25;7(1):e2353667. doi: 10.1001/jamanetworkopen.2023.53667 (PMC10811560; doi:10.1001/jamanetworkopen.2023.53667)
Supplement: Supplement 1. — eMethods. Exposures and Outcomes in Study Population eReferences. [file jamanetwopen-e2353667-s001.pdf]

Supplementary Online Content

Michelson KA, Bachur RG, Rangel SJ, Finkelstein JA, Monuteaux MC, Goyal MK. Disparities in diagnostic timeliness and outcomes of pediatric appendicitis. *JAMA Netw Open*. 2024;7(1):e2353667. doi:10.1001/jamanetworkopen.2023.53667

eMethods. Exposures and Outcomes in Study Population
eReferences.

This supplementary material has been provided by the authors to give readers additional information about their work.

© 2024 Michelson KA et al. *JAMA Network Open*.

eMethods. Exposures and Outcomes in Study Population

Identifying patients with appendicitis

Children with appendicitis were identified using International Classification of Diseases, 9th and 10th Editions, Clinical Modification (ICD-9-CM and ICD-10-CM) codes (ICD-9-CM 540-542; ICD-10-CM K35-K37). Each child's first diagnosis in the database was included. We excluded children transferred with missing data for the receiving hospital, those hospitalized within a week prior to diagnosis, and children with no longitudinal identifier that would allow identification of multiple encounters for an individual.

Delayed diagnosis outcome

The outcome was a probable delayed diagnosis of appendicitis using a validated algorithm that detects delays using administrative data, and was previously applied to evaluate hospital factors associated with delayed diagnosis.[1,2] All potentially delayed diagnoses had two ED encounters within 7 days with the second encounter resulting in a diagnosis of appendicitis. The algorithm assigns a probability of delay for each patient based on the interval between encounters, initial encounter diagnoses, presence of perforation at diagnosis, and other factors. The algorithm was derived and validated in distinct cohorts of children and the predicted probabilities of delay were shown to be accurate.

We defined a probable delay as a likelihood of delay ≥ 75%. Children with one ED encounter or those with a delay likelihood < 75% were considered to have a timely diagnosis. The index encounter was defined as the initial encounter for patients with delayed diagnosis and the diagnosis encounter for those with timely diagnosis. This represented the earliest opportunity to diagnose appendicitis.

Variables

The co-primary exposures were race/ethnicity ("race") and area-level socioeconomic status. Race was defined as Asian or Pacific Islander (API), NH-Black, Hispanic, NH-White, or Other, and was determined from the data source. Socioeconomic status was defined using the Child Opportunity Index (COI) of the patient's ZIP code.[3] The COI is an area-level measure that measures a range of factors that have been associated with child opportunity, encompassing domains of education, health/environment, and social/economic. COI is reported as a percentile nationally and we categorized it by quartile (quartile 1: lowest opportunity, quartile 4: highest). Race and COI were carried forward from the most recent prior encounter if missing.

Identifying appendicitis complications

Hospital length of stay was obtained directly from the Healthcare Cost and Utilization Project (HCUP) databases. Intensive care unit hospitalization was ascertained from the HCUP charge file based on state-specific line-item revenue center codes for intensive care. Perforated appendicitis was measured using previously validated diagnosis codes[4] (ICD-9-CM 540.0-540.1; ICD-10-CM K35.2, K35.20-K35.21, K35.32-K35.33). Abdominal abscess drainage was determined based on the presence of a compatible procedure code (CPT 44900; ICD-9-PCS 47.2, 54.91, 97.82; ICD-10-PCS 0D9630Z,

© 2024 Michelson KA et al. *JAMA Network Open*.

0D963ZZ, 0D9H30Z, 0D9J0ZZ-0D9J30Z, 0D9J3ZZ-0D9J40Z, 0D9J4ZZ-0D9J70Z, 0D9J7ZZ-0D9J80Z, 0D9P30Z, 0D9W30Z, 0D9W3ZZ-0D9W40Z, 0D9W4ZZ, 0F9G30Z, 0W9F30Z, 0W9F3ZZ-0W9F40Z, 0W9F4ZZ, 0W9G30Z-0W9G40Z, 0W9G4ZZ, 0W9H30Z, 0W9H3ZZ, 0W9J30Z, 0W9J3ZZ, 0WPGX0Z). Bowel resection was defined as the presence of a compatible procedure code (ICD-9-PCS 17.31-39, 45.61-63, 45.71-76, 45.79, 45.81-83, 46.02; ICD-10-PCS 0DB80ZZ, 0DB84ZZ, 0DBB0ZZ, 0DBB4ZZ, 0DBC4ZZ, 0DBE0ZZ, 0DBF0ZZ, 0DBH0ZZ, 0DBH4ZZ, 0DBH8ZZ, 0DBK4ZZ, 0DBM4ZZ, 0DBN0ZZ, 0DT80ZZ, 0DTA0ZZ, 0DTB0ZZ, 0DTC0ZZ, 0DTF0ZZ-0DTF4ZZ, 0DTH0ZZ-0DTH4ZZ, 0DTM4ZZ). The number of abdominal surgeries was counted as the number of unique hospital days on which an abdominal procedure was billed. Abdominal surgeries were defined using procedure codes (CPT 44x, 49x; ICD-9-PCS 47.x, 54.x; ICD-10-PCS 0Dx, 0W*Gx, 0W*Hx, 0W*Ix, 0W*Jx; *=any single character; x=any string of characters). Sepsis was determined from diagnosis codes (ICD-9-CM 785.52, 995.91-92; ICD-10-CM A02.1, A03.9, A20.7, A21.7, A22.7, A23.9, A24.1, A26.7, A28.0, A28.2, A32.7, A39.2-A39.4, A40, A41, A42.7, B00.7, P35.2, P36, P37.2, P37.5, R65.2).

eReferences.

1. Michelson KA, Bachur RG, Dart AH, et al. Identification of delayed diagnosis of paediatric appendicitis in administrative data: a multicentre retrospective validation study. *BMJ Open*. 2023;13(2):e064852. doi:10.1136/bmjopen-2022-064852
2. Michelson KA, Bachur RG, Rangel SJ, Monuteaux MC, Mahajan P, Finkelstein JA. Emergency Department Volume and Delayed Diagnosis of Pediatric Appendicitis: A Retrospective Cohort Study. *Ann Surg*. 2023;In Press.
3. Acevedo-Garcia D, Noelke C, McArdle N, et al. Racial And Ethnic Inequities In Children's Neighborhoods: Evidence From The New Child Opportunity Index 2.0. *Health Aff*. 2020;39(10):1693-1701. doi:10.1377/hlthaff.2020.00735
4. Michelson KA, Dart AH, Finkelstein JA, Bachur RG. Validation of an Automated System for Identifying Complications of Serious Pediatric Emergencies. *Hosp Pediatr*. 2021;11(8):864-878. doi:10.1542/hpeds.2020-005792

© 2024 Michelson KA et al. *JAMA Network Open*.
