# Supplementary material for: Disparities in Diagnostic Timeliness and Outcomes of Pediatric Appendicitis
Source: JAMA Netw Open. 2024 Jan 25;7(1):e2353667. doi: 10.1001/jamanetworkopen.2023.53667 (PMC10811560; doi:10.1001/jamanetworkopen.2023.53667)
Supplement: Supplement 2. — Data Sharing Statement [file jamanetwopen-e2353667-s002.pdf]

Data Sharing Statement

Michelson. Disparities in Diagnostic Timeliness and Outcomes of Pediatric Appendicitis. *JAMA Netw Open*. Published January 25, 2024. doi:10.1001/jamanetworkopen.2023.53667

Data
Data available: No

Additional Information
Explanation for why data not available: The data are publicly available from HCUP but we are unable to share it per our data use agreement with the data supplier. We will share our analysis code in R upon reasonable request.
